# Acute Mesenteric Ischemia Secondary to Candida Endocarditis: A Case Report

**DOI:** 10.7759/cureus.56747

**Published:** 2024-03-22

**Authors:** Tatsuya Ochi, Shota Kikuta, Takeshi Nishimura, Satoshi Ishihara

**Affiliations:** 1 Department of Emergency and Critical Care Medicine, Hyogo Emergency Medical Center, Kobe, JPN

**Keywords:** case report, infective endocarditis, candida endocarditis, candida tropicalis, acute mesenteric ischemia (ami)

## Abstract

A 36-year-old man presented with abdominal pain, vomiting, and diarrhea. On arrival, his vital signs were remarkable for fever (39.3 °C) and tachycardia (127 beats/min, regular). His abdomen was distended, and a blood test showed elevations of inflammatory markers. Contrast-enhanced computed tomography revealed a superior mesenteric artery thrombus, ischemic colitis, ascites, and infarctions in the spleen and right kidney. He was diagnosed with bowel necrosis due to acute mesenteric ischemia (AMI). An emergent laparotomy was performed. The following day, *Candida tropicalis* was identified in the blood culture. In addition, transthoracic echocardiography revealed vegetation on the mitral valve leaflet. These findings were suggestive of infective endocarditis (IE) caused by *C. tropicalis* (*Candida* endocarditis); thus, the patient underwent surgical mitral valve replacement with the administration of antifungal therapy. Following postoperative intensive care and a prolonged course of antifungal treatment, he achieved a full recovery. AMI is only rarely caused by IE, and this case is the first reported instance of AMI secondary to* Candida* endocarditis. When encountering patients with AMI without any risk factors for thromboembolism, clinicians should be aware that IE may cause AMI.

## Introduction

Acute mesenteric ischemia (AMI), characterized by sudden hypoperfusion in the small intestine, is a life-threatening condition [1,2]. The etiologies of AMI include embolic, thrombotic, and non-obstructive causes, with embolic mechanisms being the most common [1,2]. In rare instances, mesenteric embolism and AMI can result from infective endocarditis (IE) [3,4]. Such cases require prompt diagnosis and comprehensive treatment involving medical, surgical, and cardiothoracic interventions [3]. Nevertheless, due to its rarity, literature on AMI associated with IE is scarce, leaving optimal management uncertain. We present a unique and instructive case of AMI complicated by *Candida* endocarditis.

## Case presentation

**Figure 1:**
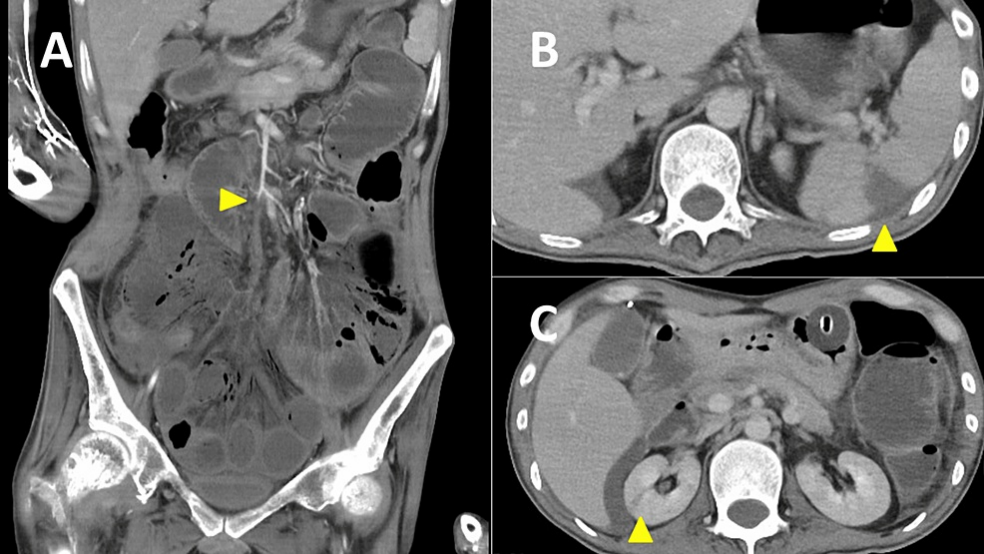
Contrast-enhanced computed tomography showing a superior mesenteric artery thrombus, ischemic colitis, ascites, and infarctions in the spleen and right kidney.

A 36-year-old man was transferred to our hospital due to fever, vomiting, and diarrhea. He had previously been treated for* Candida*-associated central venous catheter infection with catheter removal and antifungal therapy, which was completed three months prior. Upon arrival, his vital signs were as follows: blood pressure of 111/66 mmHg, heart rate of 127 beats/min and regular, respiratory rate of 21 breaths/min, and body temperature of 39.3 °C. Physical examination revealed abdominal distension. An electrocardiogram showed sinus tachycardia. The laboratory findings were significant, indicating increases in neutrophil-dominant leukocytosis, anemia, thrombocytopenia, elevated C-reactive protein levels, and coagulation abnormalities (Table 1). Contrast-enhanced computed tomography conducted at the referring hospital revealed thrombosis at the superior mesenteric artery (SMA), accompanied by small bowel ischemia, ascites, and infarctions in the spleen and right kidney (Figures 1A-1C).

**Table 1:** Initial laboratory results on admission /mm^3^ = per 0.001 milliliter; g/dL = gram per deciliter; mg/dL = milligram per deciliter; µg/mL = microgram per milliliter

Laboratory Parameters	Patient's values	Reference range
White blood cell (WBC)	24,700/mm^3^	3,300-8,600/mm^3^
Neutrophils	93.5%	32.0-75.0%
Lymphocytes	2.6%	18.0-47.0%
Monocytes	3.7%	2.0-8.0%
Eosinophils	0.1%	< 7%
Basophils	0.1%	< 1%
Hemoglobin	8.8 g/dL	13.7-16.8 g/dL
Platelet	115,000/mm^3^	158,000-348,000/mm^3^
Serum albumin	2.0 g/dL	3.8-5.1 g/dL
C-reactive protein	40.69 mg/dL	< 0.3 mg/dL
Fibrinogen	> 700 mg/dL	200 - 400 mg/dL
Prothrombin time (PT)	15.9 seconds	10.0-13.5 seconds
Activated partial thromboplastin time (APTT)	87.2 seconds	24.0-39.0 seconds
Fibrin degradation products (FDP)	15.6 µg/mL	< 5.0 µg/mL
D-dimer	< 1.0 µg/mL	< 1.0 µg/mL

**Figure 2:**
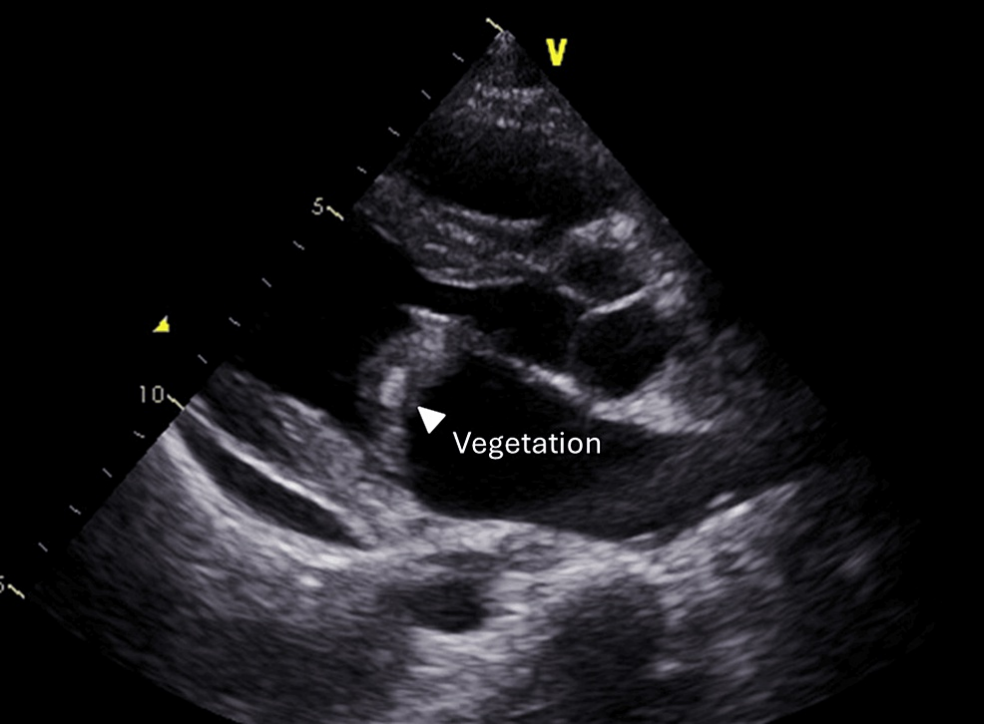
Transthoracic echocardiography showing vegetation on the mitral valve leaflet.

Based on these findings, the patient was diagnosed with bowel necrosis due to AMI. An emergent laparotomy was performed. During surgery, necrosis of the ileum and impaired palpation of the ileocolic artery were observed, leading to a procedure involving resection of the small intestine and ileocecal region. The following day, two sets of blood cultures drawn upon the patient’s arrival yielded positive results for* Candida* species, subsequently identified as *Candida tropicalis*. In addition, transthoracic echocardiography revealed mitral valve vegetation measuring 23 × 13 mm (Figure 2).

These findings suggested IE due to* C. tropicalis* (*Candida* endocarditis); therefore, the patient underwent surgical mitral valve replacement and initiation of antifungal therapy. A heart valve culture identified *C. tropicalis*, which was the same organism grown from the blood cultures. Screening tests did not reveal a condition resulting in a hypercoagulable state, such as protein C/S deficiency, antithrombin III deficiency, or antiphospholipid antibody syndrome. The patient received postoperative intensive care and was administered six weeks of intravenous antifungal agent (Micafungin) followed by three months of chronic suppressive therapy (oral Voriconazole), resulting in complete resolution without complications.

## Discussion

The presented case exemplifies the diagnostic complexity associated with IE, particularly when it presents atypical manifestations such as AMI, thereby complicating its recognition. IE often arises due to complications from septic embolism, which may mimic localized ischemia, bleeding, organ failure, infections, and even systemic vasculitis [5,6]. Consequently, the diagnosis of IE tends to be delayed, which can lead to poorer outcomes and increased mortality [7].

Patients with IE are well known to be at increased risk of arterial thromboembolic events [8,9]. However, the occurrence of AMI as a complication, as observed in this case, is uncommon. In a previous report of 68 IE patients, 35 experienced embolic complications, of which only one involved mesenteric artery embolization [4]. Moreover, instances of *Candida* endocarditis complicating AMI are unprecedented in the literature, underscoring the uniqueness of the present case.

On the patient’s day of admission, we identified no apparent risk factors for embolic AMI, such as a history of arrhythmias, valvular disease, or coronary heart disease [10], leaving its cause unknown. Fortunately, the following day, the growth of *Candida* spp. was identified from blood cultures obtained upon admission, prompting consideration of* Candida* endocarditis as the cause of AMI. A subsequent transthoracic echocardiogram confirmed the diagnosis of *Candida* endocarditis, leading to definitive therapy including mitral valve replacement surgery and initiation of antifungal therapy. This clinical course implies the importance of considering diagnostic testing for IE, such as blood culture and echocardiography, when encountering AMI in the absence of other common etiologies.

## Conclusions

This case highlights an exceedingly rare occurrence of AMI due to *Candida* endocarditis. It suggests the necessity for clinicians to consider IE as a potential cause of AMI where the etiology is unclear and common causes of embolism are not evident. We believe that these findings will contribute to an appropriate approach to similar cases in the future.

## References

[1] Chou EL, Wang LJ, McLellan RM (2021). Evolution in the presentation, treatment, and outcomes of patients with acute mesenteric ischemia. Ann Vasc Surg.

[2] Nuzzo A, Maggiori L, Ronot M (2017). Predictive factors of intestinal necrosis in acute mesenteric ischemia: prospective study from an intestinal Stroke Center. Am J Gastroenterol.

[3] Quek E, Monkman B, Madani Y (2022). Lessons of the month 1: mesenteric ischaemia secondary to infective endocarditis. Clin Med (Lond).

[4] Millaire A, Leroy O, Gaday V (1997). Incidence and prognosis of embolic events and metastatic infections in infective endocarditis. Eur Heart J.

[5] Delgado V, Ajmone Marsan N, de Waha S (2023). 2023 ESC guidelines for the management of endocarditis. Eur Heart J.

[6] Shi XD, Li WY, Shao X, Qu LM, Jiang ZY (2020). Infective endocarditis mimicking ANCA-associated vasculitis: does it require immunosuppressive therapy?: a case report and literature review. Medicine (Baltimore).

[7] Kreitmann L, Montaigne D, Launay D (2020). Clinical characteristics and outcome of patients with infective endocarditis diagnosed in a Department of Internal Medicine. J Clin Med.

[8] Shapiro S, Kupferwasser LI (2001). Echocardiography predicts embolic events in infective endocarditis. J Am Coll Cardiol.

[9] Mangoni ED, Adinolfi LE, Tripodi M-F (2003). Risk factors for "major" embolic events in hospitalized patients with infective endocarditis. Am Heart J.

[10] Liao G, Chen S, Cao H, Wang W, Gao Q (2019). Review: Acute superior mesenteric artery embolism: a vascular emergency cannot be ignored by physicians. Medicine (Baltimore).

